# PET/MR fusion texture analysis for the clinical outcome prediction in soft-tissue sarcoma

**DOI:** 10.1186/s40644-021-00438-y

**Published:** 2022-01-12

**Authors:** Wenzhe Zhao, Xin Huang, Geliang Wang, Jianxin Guo

**Affiliations:** Department of Radiology, the First Affiliated Hospital of Xi’an Jiaotong University, Xi’an, 710061 Shaanxi province PR China

**Keywords:** Soft-tissue sarcoma (STS), PET/MR fusion, Texture analysis

## Abstract

**Background:**

Various fusion strategies (feature-level fusion, matrix-level fusion, and image-level fusion) were used to fuse PET and MR images, which might lead to different feature values and classification performance. The purpose of this study was to measure the classification capability of features extracted using various PET/MR fusion methods in a dataset of soft-tissue sarcoma (STS).

**Methods:**

The retrospective dataset included 51 patients with histologically proven STS. All patients had pre-treatment PET and MR images. The image-level fusion was conducted using discrete wavelet transformation (DWT). During the DWT process, the MR weight was set as 0.1, 0.2, 0.3, 0.4, …, 0.9. And the corresponding PET weight was set as 1- (MR weight). The fused PET/MR images was generated using the inverse DWT. The matrix-level fusion was conducted by fusing the feature calculation matrix during the feature extracting process. The feature-level fusion was conducted by concatenating and averaging the features. We measured the predictive performance of features using univariate analysis and multivariable analysis. The univariate analysis included the Mann-Whitney U test and receiver operating characteristic (ROC) analysis. The multivariable analysis was used to develop the signatures by jointing the maximum relevance minimum redundancy method and multivariable logistic regression. The area under the ROC curve (AUC) value was calculated to evaluate the classification performance.

**Results:**

By using the univariate analysis, the features extracted using image-level fusion method showed the optimal classification performance. For the multivariable analysis, the signatures developed using the image-level fusion-based features showed the best performance. For the T1/PET image-level fusion, the signature developed using the MR weight of 0.1 showed the optimal performance (0.9524(95% confidence interval (CI), 0.8413–0.9999)). For the T2/PET image-level fusion, the signature developed using the MR weight of 0.3 showed the optimal performance (0.9048(95%CI, 0.7356–0.9999)).

**Conclusions:**

For the fusion of PET/MR images in patients with STS, the signatures developed using the image-level fusion-based features showed the optimal classification performance than the signatures developed using the feature-level fusion and matrix-level fusion-based features, as well as the single modality features. The image-level fusion method was more recommended to fuse PET/MR images in future radiomics studies.

**Supplementary Information:**

The online version contains supplementary material available at 10.1186/s40644-021-00438-y.

## Introduction

Radiomics referred to the extraction high-dimensional quantitative image features from multi-modality medical images [1]. Further, these data could be used to support the decision making of precision medicine [1–3]. Several studies have been reported using radiomics method for disease diagnosis, treatment outcome assessment, and prognosis evaluation [4–7]. A study of Zhang et al. demonstrated that three-dimensional quantitative features of T2-weighted MR images could be used as candidate biomarkers for preoperative prediction of histopathological grades in patients with soft-tissue sarcoma (STS) [7]. Nie et al. found that multiparametric MR features could predict pathologic response after preoperative chemoradiation therapy in locally advanced rectal cancer. Spraker et al. found that radiomics features of MR images could be used to predict overall survival in STS [5]. Vallières et al. developed a joint PET and MR texture-based model for the early risk assessment of lung metastasis in STS [6]. Vallières et al. also found that the image features extracted based on the fusion of PET and MR images showed better classification capability than the features based on the PET or MR images alone [6].

In nowadays radiomics studies, more and more multi-modality images were used. Although multi-modality images indicated more valuable information, it was yet to be decided whether simply concatenate the multi-modality image features or fuse the multi-modality images to generate new image features. In the present studies, the feature concatenation and feature average methods were the most commonly used method for multi-modality fusion [8–10]. In addition, several studies attempted to integrate multi-modality images via the image fusion method [11, 12]. Riyahi et al. adopted the weighted summation method of normalized PET and CT images to generate a single fused PET-CT image [11]. Zhou et al. investigated the possibility of using features extracted based on the wavelet-based fusion images of PET and MR to predict the progression of patients with mild cognitive impairment to Alzheimer’s disease [12]. Different from the researches working on feature-level fusion and image-level fusion, Parekh et al. proposed a novelty fusion method of designing a signature co-occurrence matrix to merge matrices constructed from multiparametric MR images [13]. They concluded that the features extracted based on multi-parametric fusion matrices showed better performance for the diagnosis of breast cancer and brain stroke compared to the features extracted based on single parameter images.

Various fusion strategies (feature-level fusion, matrix-level fusion, and image-level fusion) might lead to different feature values, which might ultimately affect the classification performance of the image features and proposed models. To the best of our knowledge, the classification performance of features based on different fusion levels of PET and MR images has not been fully investigated and compared in the current radiomics studies. In this study, we used multi-level fusion strategies to fuse PET and MR images in patients with STS. Further, we measured the classification capability of features extracted based on different fusion methods of PET and MR images.

## Methods

### Study design

**Fig. 1:**
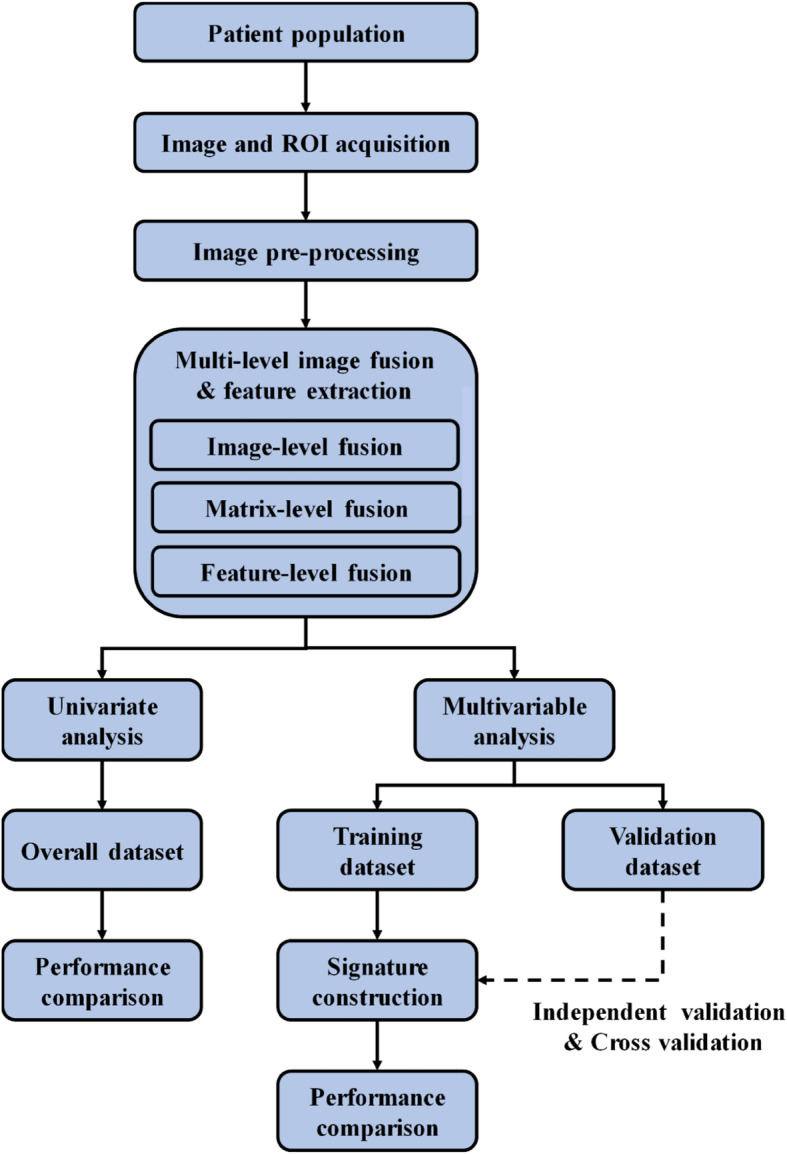
The workflow for this study

The workflow of this study was presented in Fig. 1. This study included four major parts: (i) image and region of interest (ROI) acquisition, (ii) image pre-processing, (iii) multi-level image fusion and feature extraction, and (iv) statistical analysis.

### Image and region of interest acquisition

#### Image acquisition

The dataset used in this study was from The Cancer Imaging Archive (https://wiki.cancerimagingarchive.net/display/Public/Soft-tissue-Sarcoma), containing FDG-PET/MR imaging data and clinical data of 51 patients with histologically proven STS in the extremities. The clinical outcome of this study was tumor recurrence/ metastasis. Of the 51 patients, 27 patients (52.94%) showed the tumor recurrence/ metastasis, and 24 patients (47.06%) showed no recurrence/ metastasis.

For the overall patient dataset, each patient image dataset was already numbered, as STS-001, STS-002, STS-003, …, STS-051. We separated the dataset as a training dataset and independent validation dataset according to the patient number at an approximate ratio of 3:1 according to the previous studies [14, 15]. As a result, we used the patient dataset numbered between 001 and 038 as the training dataset, and the dataset numbered between 039 and 051 as the independent validation dataset. For the training dataset, 21 patients underwent tumor recurrence/ metastasis, and 17 patients had no tumor recurrence/ metastasis. And for the validation dataset, 7 patients had tumor recurrence/ metastasis, and 6 patients had no tumor recurrence/ metastasis. To measure the possible bias during the separation, we used the demographic comparison between the training and validation dataset. There were no significant differences in the tumor recurrence/ metastasis ratio between the training and validation datasets (*P* = 0.8056, Chi-squared test), demonstrating no bias introduced into the study during the separation and justifying their usage as the training and validation datasets. The tumor volume was 479.24 ± 510.86 cm^3^ (Range: 16.67–2313.96 cm^3^) for the training dataset and 402.59 ± 321.04 cm^3^ (Range: 33.01–1043.40 cm^3^) for the validation dataset. No significant difference was observed in tumor volume between these two datasets (*P* = 0.8968, Chi-squared test).

Three types of MR sequences were selected for this study, namely T1-weighted, fat-saturated T2-weighted, and short tau inversion recovery sequences. For the T1-weighted MR imaging, the median in-plane resolution was 0.74× 0.74 mm^2^ (range: 0.23–1.64 mm^2^), the median slice thickness was 5.5 mm (range: 3.0–10.0 mm). For the fat-saturated T2-weighted MR imaging, the median in-plane resolution was 0.63× 0.63 mm^2^ (range: 0.23–1.64 mm^2^), the median slice thickness was 5.0 mm (range: 3.0–8.0 mm). For the short tau inversion recovery MR imaging, the median in-plane resolution was 0.86× 0.86 mm^2^ (range: 0.23–1.72 mm^2^), the median slice thickness was 5.0 mm (range: 3.0–10.0 mm). The fat-saturated T2-weighted images were selected by default for the higher axial scan availability. When fat-saturated T2-weighted images were not available, the short tau inversion recovery images were used.

For the FDG-PET imaging, the contrast was injected intravenously (range: 210–620 MBq). Approximately 60 mins following the injection, the whole-body PET images were acquired. The in-plane resolution was 5.47× 5.47 mm^2^ (range: 3.91–5.47 mm), the slice thickness was 3.27 mm for all patients.

#### Region of interest acquisition

**Fig. 2:**
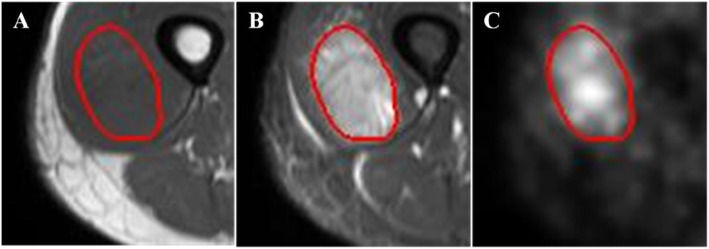
An example of tumor delineation (red line) in PET and MR images of a patient with STS. **A** T1-weighted MR images; **B** T2-weighted MR images; **C** PET images

All images were reviewed in the MIM® software (MIM Software Inc., Cleveland, OH) by an experienced radiation oncologist. The 3D region of interest (ROI) was manually delineated on the T2-weighted MR images. Then, the ROI was propagated to PET images and T1-weighted MR images using rigid registration based on MIM®. An example of 2D ROI in PET and MR images of a patient with STS was provided in Fig. 2.

### Imaging pre-processing

Before the texture feature extraction process, the PET and MR DICOM images were transferred into the MATLAB software (version R2019b; The MathWorks Inc., Natick, MA). The PET images were first converted to standard uptake value (SUV) maps, and followed by the operation of the square-root transform to stabilize the noise in the PET images. For the MR images, to make the feature extraction process more reliable, we rejected the voxels within the tumor volumes with intensities outside the range of μ ± 3σ according to the suggestion of Collewet et al. [16]. Since image feature values were sensitive to variations of the voxel size, the widely changing in voxel size might eliminate the robustness and reproducibility of the feature extraction process. Thus, we performed the image resampling before the feature extraction process to keep isotropic voxel sizes to be rotationally invariant. The image resampling also allowed the comparison between analyses from different samples or datasets. The image resampling was conducted using the linear interpolation method with the isotropic voxel size of 1.0 × 1.0 × 1.0 mm^3^. In addition, the grey level was discretized to the fixed bin size of 64 to make features tractable according to the previous studies [17–19].

### Multi-level PET/MR fusion

For the purpose of characterizing the tumor volume more comprehensively, we used the multi-level fusion strategies to fuse PET and MR images in patients with STS, including the image-level fusion, matrix-level fusion, and feature-level fusion.

#### Image-level PET/MR fusion

We fused the PET images and MR images into a single fused image to enhance the texture information. The most commonly used image fusion algorithms of the 3D discrete wavelet transform (DWT)-based image fusion was adopted in this study [6, 20]. The DWT-based image fusion method was suitable for multi-modality images.

The DWT-based image fusion method firstly decomposed the PET images and MR images to the same decomposition level using the wavelet basis function *symlet8* according to the previous studies [6, 20]. Then, we combined the wavelet coefficients of PET and MR sub-bands using the weighted average method [6, 20]. The weight of MR images (denoted as MR weight) was set as 0.1, 0.2, 0.3, 0.4, …, 0.9. And the corresponding PET weight was set as 1- (MR weight). Finally, we reconstructed the PET/MR fusion images using the 3D inverse DWT. The detailed descriptions and software code on MATLAB could be achieved by https://github.com/mvallieres/radiomics.

#### Matrix-level PET/MR fusion

For the texture feature extraction, the texture feature matrix was generated based on the distributions of the center voxel with surrounding voxels. The texture feature was calculated based on the generated calculation matrix. For the matrix-level PET/MR fusion, two feature texture matrices were constructed based on PET and MR images, respectively. Then, we merged the two matrices into a single fused matrix. The fused texture matrix considered the voxel distributions within tumor volumes in PET images and MR images simultaneously. Further, the texture features were calculated based on the fused texture matrices.

#### Feature-level PET/MR fusion

The most commonly and simply used feature fusion method was feature concatenation [10, 21, 22]. This method simply connected the image features of different modalities. The number of connected features equaled to the sum of the number of PET features and MR features. In addition to the feature concatenation method, we also used the feature average method to investigate whether it is useful for classification. The number of average features equaled to the number of PET and MR features.

### Quantitative feature extraction

A total of 136 quantitative image features were extracted for the single modality images, image-level fusion method, and matrix-level fusion method. The texture features were divided into six families in this study, including grey level co-occurrence matrix (GLCM)-based features, grey level run length matrix (GLRLM)-based features, grey level size zone matrix (GLSZM)-based features, grey level distance zone matrix (GLDZM)-based features, neighborhood grey tone difference matrix (NGTDM)-based features, and neighboring grey level dependence matrix (NGLDM)-based features [8, 23]. The feature extraction process was conducted according to the Standardized Environment for Radiomics Analysis (SERA) package on MATLAB software (version R2019b; The MathWorks Inc., Natick, MA) [23]. The package complied with the imaging biomarker standardization initiative (IBSI) guidelines [24]. The feature names and abbreviations used in the study were provided in Supplementary Material 1.

### Statistical analysis

#### Univariate analysis for image feature

We used the univariate analysis to assess the classification performance of image features by using the Mann–Whitney U test and receiver operating characteristic (ROC) analysis. A *P value* < 0.05 was considered statistically significant. The area under the ROC curve (AUC) value was calculated to evaluate the classification performance. We also measured the correlation between the T1-weighted MR image-based features, T2-weighted MR image-based features, and multi-level fusion-based features by using the Pearson correlation analysis. The features with correlation coefficients greater than 0.8 were considered as significant correlations [25].

#### Multivariable analysis using independent validation

We used the multivariable analysis to assess the classification performance of signatures. Prior to developing image signatures, we used the Z-score method to normalize the image features in the training dataset. The features of the validation dataset were normalized using the mean and standard deviation values calculated based on the training dataset. The signature was developed based on the training dataset using the features selected by the maximum relevance minimum redundancy (mRMR) approach, which was suitable to select the most optimal image features from high-dimensional data. The mRMR could rank the features according to the importance of the classification label and redundancy to other features. Thus, we could select the top features to establish the signature to avoid overfitting. Based on the rule of thumb, the ratio of the sample size to the number of predictor variables should be at least 10:1 [26]. In this study, the potential feature number was limited to 4 to establish the signature.

#### Multivariable analysis using cross validation

The patient dataset used in this study was relatively small. This might produce unavoidable statistical bias during the signature construction and validation processes. In this current study, the 4-fold cross validation method was also used. The overall patient cohort was randomly separated into four partitions, with three partitions used as the temporary training set and the remaining one as the temporary validation set. The radiomics signature was developed based on the temporary training set and validated based on the temporary validation set. The method to build the radiomics signature was the same as the methods used in the previous descriptions. The signature training and validation process were conducted 4 times, and the mean performance with standard deviation was reported as the 4-fold cross validation performance. All statistical analysis used in this study was conducted using R software (version 3.51).

## Results

### Univariate analysis for image feature

By using the univariate analysis for image features based on single modality images, the image features based on PET images showed better classification performance than the features based on the T1-weighted MR images and T2-weighted MR images. For the features based on PET images, a number of 79 features showed significant classification capability for the tumor recurrence/ metastasis prediction. The mean AUC value was 0.7254 ± 0.0366 for these PET features. By contrast, a number of 52 T1-weighted MR image-based features and 71 T2-weighted MR image-based features showed significant predictive performance in the tumor recurrence/ metastasis prediction. The mean AUC value was 0.7196 ± 0.0340 and 0.6985 ± 0.0228 for these T1-weighted MR image-based features and T2-weighted MR image-based features, respectively.

**Fig. 3:**
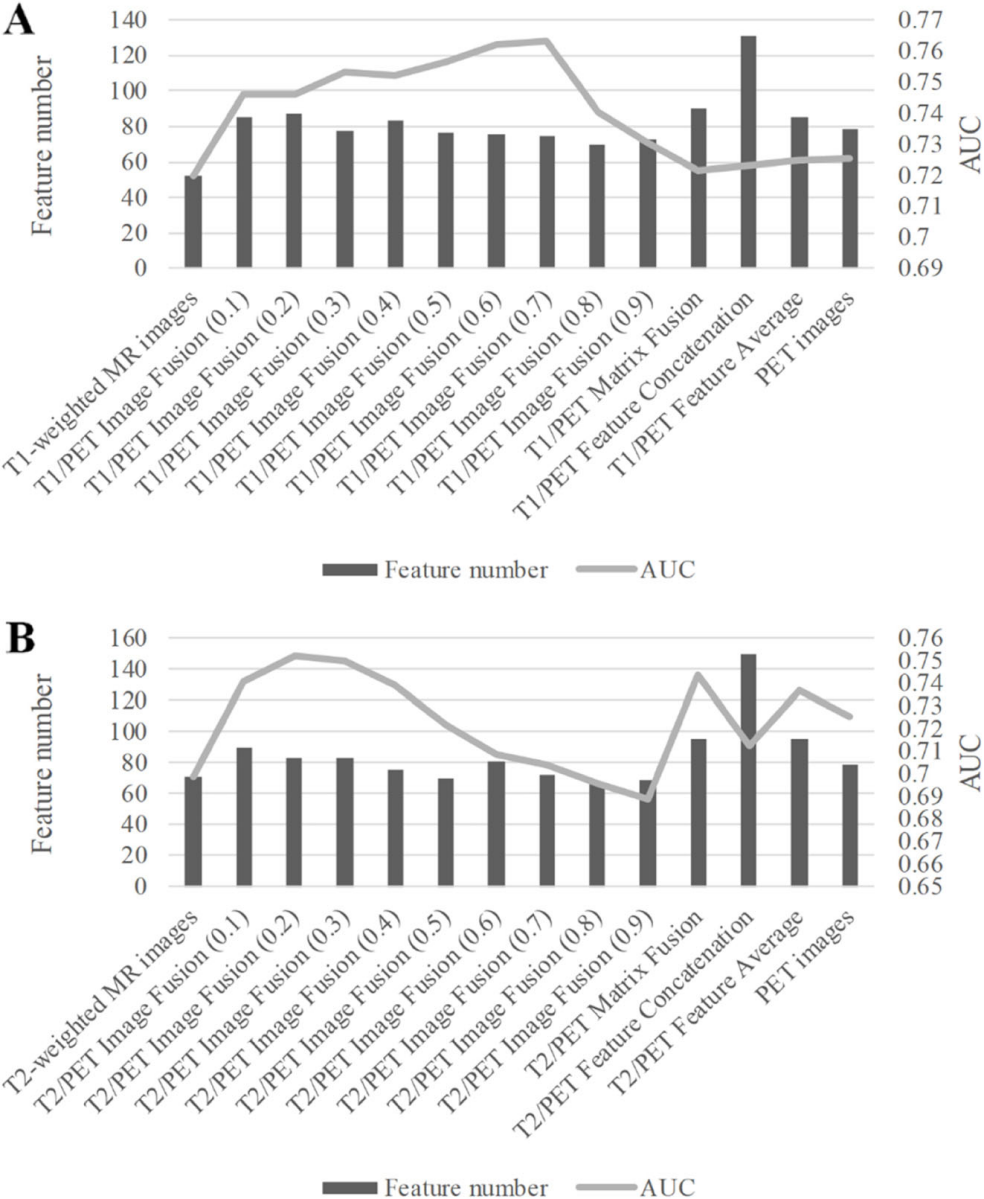
Univariate analysis for imaging feature based on different image fusion methods. **A** T1-wighted MR images and PET images; **B** T2-wighted MR images and PET images. The line chart indicated the number of significant imaging features. The bar chart indicated the mean AUC value of the significant image features

For the image-level PET/MR fusion, we observed that more features showed significant predictive capacity than the PET-based and MR-based features. By using the T1/PET image-level fusion with the MR weight of 0.2, a number of 87 features were selected with the mean AUC of 0.7462 ± 0.0504. For the MR weight of 0.1 for T2/PET image-level fusion, 90 features showed significant classification capability for the tumor recurrence/ metastasis prediction with the mean AUC of 0.7411 ± 0.0442. Detailed performance of univariate analysis for all features was provided in Table 1 and Fig. 3.

**Table 1:** Detailed performance of univariate analysis for imaging feature with each modality and fusion method

Class	Feature number	AUC value
No fusion-based features		
T1-weighted MR images	52	0.7196 ± 0.0340
T2-weighted MR images	71	0.6985 ± 0.0228
PET images	79	0.7254 ± 0.0366
Image-level fusion based features		
T1/PET Image Fusion (0.1)	85	0.7459 ± 0.0414
T1/PET Image Fusion (0.2)	87	0.7462 ± 0.0504
T1/PET Image Fusion (0.3)	78	0.7533 ± 0.0463
T1/PET Image Fusion (0.4)	83	0.7523 ± 0.0420
T1/PET Image Fusion (0.5)	77	0.7567 ± 0.0386
T1/PET Image Fusion (0.6)	76	0.7619 ± 0.0448
T1/PET Image Fusion (0.7)	75	0.7631 ± 0.0432
T1/PET Image Fusion (0.8)	70	0.7407 ± 0.0338
T1/PET Image Fusion (0.9)	73	0.7306 ± 0.0365
T2/PET Image Fusion (0.1)	90	0.7411 ± 0.0442
T2/PET Image Fusion (0.2)	83	0.7519 ± 0.0438
T2/PET Image Fusion (0.3)	83	0.7503 ± 0.0420
T2/PET Image Fusion (0.4)	75	0.7392 ± 0.0328
T2/PET Image Fusion (0.5)	70	0.7215 ± 0.0283
T2/PET Image Fusion (0.6)	81	0.7084 ± 0.0244
T2/PET Image Fusion (0.7)	72	0.7041 ± 0.0226
T2/PET Image Fusion (0.8)	66	0.6957 ± 0.0195
T2/PET Image Fusion (0.9)	69	0.6885 ± 0.0167
Matrix-level fusion based features		
T1/PET Matrix Fusion	90	0.7216 ± 0.0355
T2/PET Matrix Fusion	95	0.7441 ± 0.0464
Feature-level fusion based features		
T1/PET Feature Concatenation	131	0.7231 ± 0.0359
T2/PET Feature Concatenation	150	0.7126 ± 0.0335
T1/PET Feature Average	85	0.7249 ± 0.0318
T2/PET Feature Average	95	0.7366 ± 0.0416

The number in the parentheses indicated the MR weight

For the matrix-level PET/MR image fusion, 90 T1/PET fusion-based features and 95 T2/PET fusion-based features showed significant classification capability for the tumor recurrence/ metastasis prediction. The classification performance was 0.7216 ± 0.0355 for T1/PET fusion-based features and 0.7441 ± 0.0464 for T2/PET fusion-based features.

For the feature-level PET/MR image fusion of feature average, 85 features (AUC, 0.7249 ± 0.0318) and 95 (AUC, 0.7366 ± 0.0416) features were selected from T1/PET fusion and T2/PET fusion, respectively. As for the feature concatenation-based feature fusion, the AUC value was 0.7231 ± 0.0359 by connecting the PET features and T1-weighted MR features, and 0.7126 ± 0.0335 by connecting the PET features and T2-weighted MR features.

Based on the correlation analysis, the T1-weighted and T2-weighted MR image-based features showed the highest correlation with the matrix-level fusion-based features, with the significant feature number as 81 and 72, respectively. For the image-level fusion images, with the increment of the MR weight, the correlation between image-level fusion-based features and T1-weighted or T2-weighted MR image-based features gradually increased. The detailed numbers of features with significant correlation between the T1-weighted MR image-based features, T2-weighted MR image-based features, and multi-level fusion-based features were provided in Table 2.

**Table 2:** Correlation analysis between the T1-weighted MR image-based features, T2-weighted MR image-based features and multi-level fusion-based features

Class	T1 image	T2 image
PET image	6	8
Matrix-level fusion	81	72
Feature-level fusion	68	71
Image-level fusion (0.1)	10	8
Image-level fusion (0.2)	11	8
Image-level fusion (0.3)	11	11
Image-level fusion (0.4)	11	14
Image-level fusion (0.5)	13	21
Image-level fusion (0.6)	14	56
Image-level fusion (0.7)	15	70
Image-level fusion (0.8)	48	76
Image-level fusion (0.9)	64	87

For the class column, the number in the parentheses indicated the fusion weight of the MR images. The “matrix-level fusion” indicated the T1/PET matrix fusion-based features for the correlation analysis with T1-weighted MR-based features, and the T2/PET matrix fusion-based features for the correlation analysis with T2-weighted MR-based features. The “feature-level fusion” indicated the feature average method based fusion

### Multivariable analysis using independent validation

For the signatures constructed based on single modality images, we observed the optimal performance in signatures based on PET images. The AUC value was 0.8571 (95% confidence interval (CI), 0.5732–0.9999) for the independent validation dataset. By contrast, the signatures developed based on MR images showed unfavorable accuracy (T1-weighted MR images: 0.8333 (95%CI, 0.5817–0.9999), T2-weighted MR images: 0.6904 (95%CI, 0.3792–0.9999)). The names of the features used in the signatures were provided in Supplementary Material II.

Of the three PET/MR fusion strategies, the signatures developed based on the image-level fusion-based features showed the optimal performance. For the image fusion with different MR weights, we observed diverse classification performance. For the T1/PET image-level fusion, the fusion with an MR weight of 0.1 showed the optimal performance in the validation dataset (0.9524 (95%CI, 0.8413–0.9999)). For the MR weight of 0.2, 0.4, 0.5, the signature based on the T1/PET image-level fusion all showed better performance than the signatures developed based on single modality images (MR weight of 0.2: 0.8571 (95%CI, 0.6376–0.9999); MR weight of 0.4: 0.8810 (95%CI, 0.6897–0.9999); MR weight of 0.5: 0.8571 (95%CI, 0.5771–0.9999)).

**Fig. 4:**
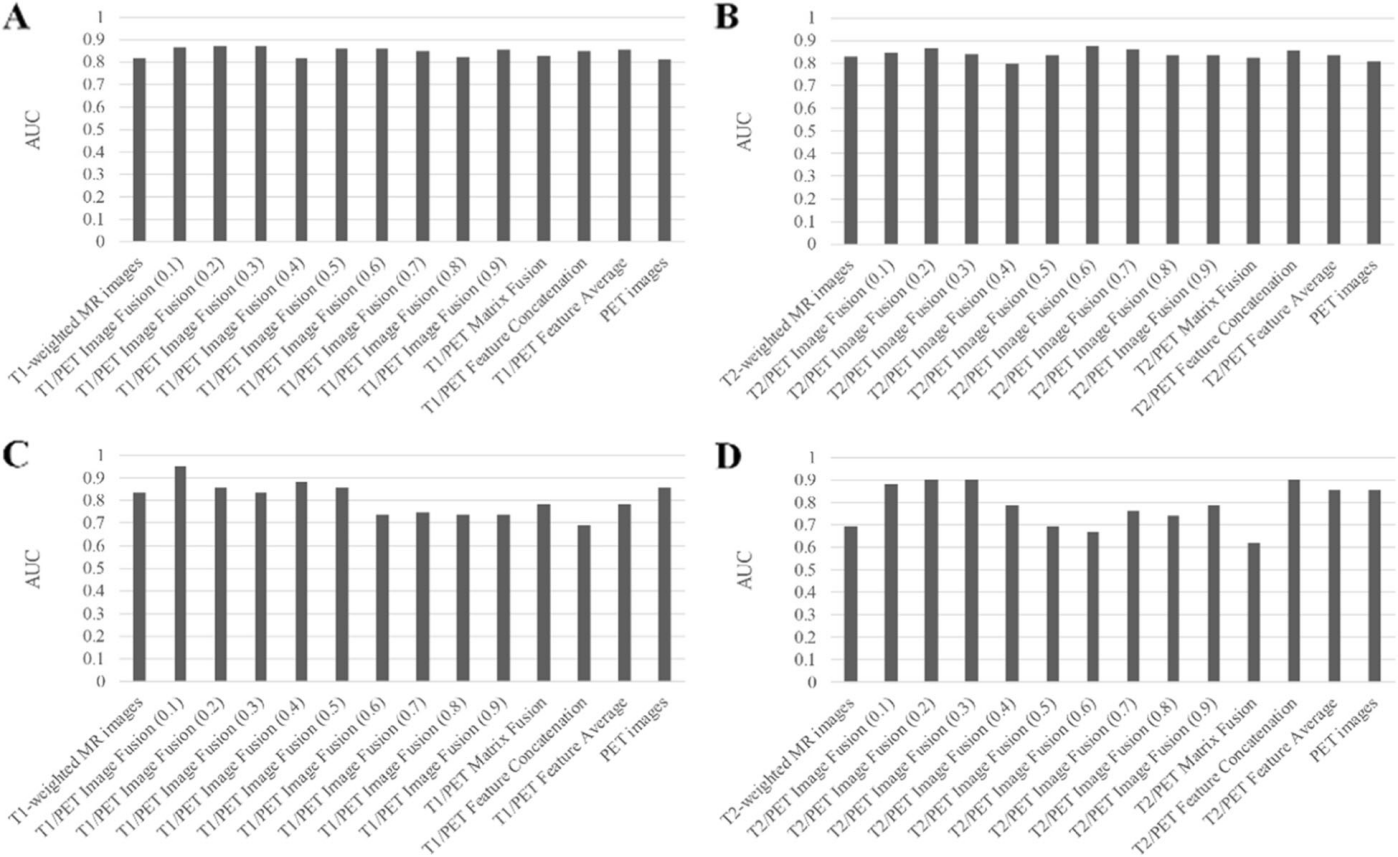
Multivariable analysis using independent validation with features based on different image fusion methods. **A** The AUC value of the signatures based on T1-wighted MR images and PET images in the training dataset; **B** the AUC value of the signatures based on T1-wighted MR images and PET images in the validation dataset; **C** the AUC value of the signatures based on T2-wighted MR images and PET images in the training dataset; **D** the AUC value of the signatures based on T2-wighted MR images and PET images in the validation dataset

For the T2/PET image-level fusion, the fusion with the MR weight of 0.3 showed the optimal performance (0.9048 (95%CI, 0.7356–0.9999)). For the MR weight of 0.1, 0.2, the signature based on the T2/PET image-level fusion showed better performance than the signatures developed based on single modality images (MR weight of 0.1: 0.8810(95%CI, 0.6897–0.9999); MR weight of 0.2: 0.9048(95%CI, 0.7090–0.9999)). The detailed performance of the signatures developed based on multi-level fusion was showed in Table 3 and Fig. 4.

**Table 3:** Performance of multivariable analysis with each modality and fusion method

Class	Training dataset	Validation dataset
No fusion-based features		
T1-weighted MR images	0.8151 (0.6809–0.9493)	0.8333 (0.5817–0.9999)
T2-weighted MR images	0.8263 (0.6849–0.9677)	0.6904 (0.3792–0.9999)
PET images	0.8095 (0.6708–0.9483)	0.8571 (0.5732–0.9999)
Image-level fusion-based features		
T1/PET Image Fusion (0.1)	0.8655 (0.7482–0.9829)	0.9524 (0.8413–0.9999)
T1/PET Image Fusion (0.2)	0.8711 (0.7606–0.9817)	0.8571 (0.6376–0.9999)
T1/PET Image Fusion (0.3)	0.8683 (0.7565–0.9802)	0.8333 (0.6897–0.9999)
T1/PET Image Fusion (0.4)	0.8179 (0.6820–0.9539)	0.8810 (0.6897–0.9999)
T1/PET Image Fusion (0.5)	0.8599 (0.7422–0.9777)	0.8571 (0.5771–0.9999)
T1/PET Image Fusion (0.6)	0.8571 (0.7340–0.9803)	0.7381 (0.4246–0.9999)
T1/PET Image Fusion (0.7)	0.8487 (0.7262–0.9712)	0.7476 (0.4898–0.9999)
T1/PET Image Fusion (0.8)	0.8207 (0.6872–0.9543)	0.7381 (0.4388–0.9999)
T1/PET Image Fusion (0.9)	0.8515 (0.7287–0.9743)	0.7381 (0.4300–0.9999)
T2/PET Image Fusion (0.1)	0.8431 (0.7200–0.9663)	0.8810 (0.6897–0.9999)
T2/PET Image Fusion (0.2)	0.8627 (0.7438–0.9817)	0.9048 (0.7090–0.9999)
T2/PET Image Fusion (0.3)	0.8403 (0.7156–0.9651)	0.9048 (0.7356–0.9999)
T2/PET Image Fusion (0.4)	0.7983 (0.6377–0.9589)	0.7857 (0.5136–0.9999)
T2/PET Image Fusion (0.5)	0.8319 (0.6913–0.9726)	0.6905 (0.3643–0.9999)
T2/PET Image Fusion (0.6)	0.8739 (0.7647–0.9832)	0.6667 (0.3398–0.9935)
T2/PET Image Fusion (0.7)	0.8571 (0.7369–0.9774)	0.7619 (0.4631–0.9999)
T2/PET Image Fusion (0.8)	0.8347 (0.7087–0.9608)	0.7381 (0.4214–0.9999)
T2/PET Image Fusion (0.9)	0.8319 (0.6996–0.9643)	0.7857 (0.5117–0.9999)
Matrix-level fusion-based features		
T1/PET Matrix Fusion	0.8291 (0.7004–0.9579)	0.7857 (0.5139–0.9999)
T2/PET Matrix Fusion	0.8235 (0.6749–0.9722)	0.6190 (0.2632–0.9749)
Feature-level fusion based features		
T1/PET Feature Concatenation	0.8459 (0.7245–0.9674)	0.6905 (0.3788–0.9999)
T2/PET Feature Concatenation	0.8543 (0.7330–0.9757)	0.9047 (0.7361–0.9999)
T1/PET Feature Average	0.8543 (0.7363–0.9723)	0.7857 (0.5139–0.9999)
T2/PET Feature Average	0.8347 (0.6963–0.9731)	0.8571 (0.6132–0.9999)

For the class column, the number in the parentheses indicated the MR weight. For the training dataset and validation dataset columns, the number in the parentheses indicated the 95% confidence interval of AUC

The matrix-level PET/MR fusion method showed the worst classification performance for both T1/PET fusion and T2/PET fusion (T1/PET fusion: 0.7857 (95%CI, 0.5139–0.9999); T2/PET fusion: 0.6190 (95%CI, 0.2632–0.9749).

For the feature-level fusion of the T1/PET images, the feature average method showed better accuracy than feature concatenation method (feature average method: 0.7857 (95%CI, 0.5139–0.9999); feature concatenation method: 0.6905 (95%CI, 0.3788–0.9999)). While for feature-level fusion of the T2/PET images, the feature average method showed worse performance than feature concatenation method (feature average method: 0.8571 (95%CI, 0.6132–0.9999); feature concatenation method: 0.9047 (95%CI, 0.7361–0.9999)).

### Multivariable analysis using cross validation

By using the 4-fold cross validation method, we observed consistent results with the independent validation method. Although the AUC value of the cross-validation and independent validation was different, the optimal signature were both developed using the image-level fusion-based features. The detailed performance of radiomics signatures using the 4-fold cross validation was provided in Supplementary Material III.

## Discussion

This study measured the classification capability of different fusion methods based on PET and MR images in patients with STS. The signatures developed using the image-level fusion-based features showed the optimal classification performance than the signatures developed using the feature-level fusion and matrix-level fusion-based features, as well as the single modality features.

We firstly compared the prediction performance of multi-level fusion features using the univariate analysis. For the single modality images-based features, the PET-based features showed better performance than the T1 and T2-based features. According to Table 1, more T1/PET multi-level fusion features showed significant classification capability than the T1-weighted MR-based features. By contrast, not all multi-level level fusion features showed more significant features than the features based on PET images. By calculating the AUC values via ROC analysis, the features based on the image-level fusion across all weighted factors showed higher AUC values than features based on the feature-level fusion and matrix-level fusion methods, as well as the single modality images. For the T2/PET multi-level fusion features, the highest mean AUC value was found using the image-level fusion features with the MR weight of 0.2. However, the number of features with significant classification capability based on the image-level fusion features with the MR weight of 0.8 and 0.9 was less than the features based on the T2-weighted MR images. The AUC values of these two fusions were also lower than the features based on the T2-weighted MR images and PET images. The optimal classification capability was observed in image-level fusion in both T1/PET and T2/PET multi-level fusions based on the univariate analysis.

By using the multivariable analysis to investigate the classification performance of signatures developed using the features based on different fusion levels, we observed consistent results with the univariate analysis. For the T1/PET fusion-based features, the image-level fusion features with the MR weight of 0.1 showed the optimal performance with an AUC of 0.9524 (95%CI, 0.8413–0.9999). While for the T2/PET fusion-based features, the optimal performance was observed using the image-level fusion with the MR weight of 0.2 and 0.3. For the image-level fusion-based features with MR weight higher than 0.3, the classification performance was all better than using T2-weighted MR-based features, nevertheless, worse than PET-based features.

For the matrix-level fusion of both T1/PET and T2/PET fusions, the developed signatures all showed worse classification performance than the corresponding T1 or T2-based features. This indicated that the classification capability of the matrix-level fusion features might be limited in STS. While for the feature-level fusion of T1/PET and T2/PET fusions, the developed signatures all showed worse classification performance than the image-level fusion-based features.

Using the fusion MR weight of 0.5 as the cutoff in the image-level fusion, an interesting behavior was observed. Based on the univariate analysis for T1/PET image-level fusion with the MR weight less than 0.5, the mean number of significant features was 83.25, and the mean AUC value was 0.7494. While for T1/PET image-level fusion with MR weight higher than 0.5, the mean number of significant features was 73.5, and the mean AUC value was 0.7491. For T2/PET image-level fusion with MR weight less than 0.5, the mean number of significant features was 82.75, while the mean AUC value was 0.7456. While for T2/PET image-level fusion with MR weight higher than 0.5, the mean number of significant features was 72, and the mean AUC value was 0.6992. The fusion factor less than 0.5 showed better classification performance than the fusion factor higher than 0.5 in both fusions. The potential reason for this behavior was that when the fusion factor was less than 0.5, the fusion features might be more correlated to PET images, meaning that the PET features were emphasized. By contrast, when the fusion factor was higher than 0.5, the fusion features might be more correlated to MR images. This reason was consistent with the results of the correlation analysis that with the increment of the MR weight in the image-level fusion process, the correlation between image-level fusion-based features and T1-weighted or T2-weighted MR image-based features gradually increased. This behavior was further confirmed based on the multivariable analysis. For T1/PET image-fusion with MR weight less than 0.5, the mean AUC value was 0.8810. While for T1/PET image-fusion with MR weight higher than 0.5, the mean AUC value was 0.7405. For T2/PET image-fusion with MR weight less than 0.5, the mean AUC value was 0.8690. While for T2/PET image-fusion with MR weight higher than 0.5, the mean AUC value was 0.7381.

In summary, we observed the better predictive performance of the image-level feature fusion method compared with features based on feature-level fusion and matrix-level fusion methods, as well as the single modality images. The similar results were consistent with several relevant studies [8, 12]. The similar results were consistent with a relevant study on PET-CT images fusion [8]. Lv et al. compared the fusion features with different fusion methods in PET and CT images [8]. They concluded that integrating information at the image level held the potential to capture more useful characteristics. Although their study was not conducted based on PET and MR images, their study demonstrated the rationality of combining multi-modality images with the image-level fusion strategy. Zhou et al. demonstrated that the wavelet-based fusion of PET and MR images prediction model showed higher prediction accuracy than the MR-based prediction model and the PET-based prediction model [12].

Many image metrics (mutual information, entropy, etc.) has been used to measure the quality of fusion images [27]. In our study, we directly measured the quality of fusion images by evaluating the predictive performance of different fusion strategies. It was interesting that the prediction performance of the image-level fusion with different weights was quite different. This phenomenon was consistent with a previous study [8].

This study still had several limitations. Firstly, the patient cohort for this study was relatively small, and only one tumor type was included in the current study. Although we conducted the independent validation and cross validation methods, the statistical bias might still be unavoidable. Our future research will be conducted with a larger patient dataset with multi-tumor types to further validate the robustness of this study. Secondly, only three commonly used feature fusion methods were included in this study. For the image-level fusion method, we only used the wavelet bead image fusion method. In the future study, we will include more feature fusion methods to find the optimal fusion method in radiomics studies.

## Conclusions

For the fusion of PET and MR images in patients with STS, the image-level fusion method showed the optimal classification performance than feature-level fusion and matrix-level fusion methods, as well as the single modality images. Thus, the image-level fusion method was more recommended to fuse PET and MR images in future radiomics studies.

## Supplementary Information

**Additional file 1.**

## Data Availability

The dataset used was downloaded from The Cancer Imaging Archive (https://wiki.cancerimagingarchive.net/display/Public/Soft-tissue-Sarcoma).

## Abbreviations

STS: Soft-tissue Sarcoma
ROC: Receiver operating characteristic
AUC: Area under the ROC curve
DWT: Discrete wavelet transformation
CI: Confidence interval
mRMR: Maximum relevance minimum redundancy
PET: Positron emission tomography
MR: Magnetic resonance
