# Supplementary material for: PET/MR fusion texture analysis for the clinical outcome prediction in soft-tissue sarcoma
Source: Cancer Imaging. 2022 Jan 12;22:7. doi: 10.1186/s40644-021-00438-y (PMC8756708; doi:10.1186/s40644-021-00438-y)
Supplement: Supplementary file 1 — Additional file 1. [file 40644_2021_438_MOESM1_ESM.docx]

***Supplementary Material***

**PET/MR fusion texture analysis for the clinical outcome prediction in soft-tissue sarcoma**

Wenzhe Zhao﹒Xin Huang﹒ Geliang Wang﹒Jianxin Guo

Ⅰ. The features extracted in the study

Ⅱ. Names of the features used in the signatures

Ⅲ. Performance of radiomics signatures using 4-flod cross validation

Ⅰ. The features extracted in the study

| **Family** | **Image feature** | **Abbreviations** |
| --- | --- | --- |
| Co-occurrence matrix (3D, averaged) | Joint maximum | cm_joint_max_3D_avg |
| Co-occurrence matrix (3D, averaged) | Joint average | cm_joint_avg_3D_avg |
| Co-occurrence matrix (3D, averaged) | Joint variance | cm_joint_var_3D_avg |
| Co-occurrence matrix (3D, averaged) | Joint entropy | cm_joint_entr_3D_avg |
| Co-occurrence matrix (3D, averaged) | Difference average | cm_diff_avg_3D_avg |
| Co-occurrence matrix (3D, averaged) | Difference variance | cm_diff_var_3D_avg |
| Co-occurrence matrix (3D, averaged) | Difference entropy | cm_diff_entr_3D_avg |
| Co-occurrence matrix (3D, averaged) | Sum average | cm_sum_avg_3D_avg |
| Co-occurrence matrix (3D, averaged) | Sum variance | cm_sum_var_3D_avg |
| Co-occurrence matrix (3D, averaged) | Sum entropy | cm_sum_entr_3D_avg |
| Co-occurrence matrix (3D, averaged) | Angular second moment | cm_energy_3D_avg |
| Co-occurrence matrix (3D, averaged) | Contrast | cm_contrast_3D_avg |
| Co-occurrence matrix (3D, averaged) | Dissimilarity | cm_dissimilarity_3D_avg |
| Co-occurrence matrix (3D, averaged) | Inverse difference | cm_inv_diff_3D_avg |
| Co-occurrence matrix (3D, averaged) | Inverse difference normalized | cm_inv_diff_norm_3D_avg |
| Co-occurrence matrix (3D, averaged) | Inverse difference moment | cm_inv_diff_mom_3D_avg |
| Co-occurrence matrix (3D, averaged) | Inverse difference moment normalized | cm_inv_diff_mom_norm_3D_avg |
| Co-occurrence matrix (3D, averaged) | Inverse variance | cm_inv_var_3D_avg |
| Co-occurrence matrix (3D, averaged) | Correlation | cm_corr_3D_avg |
| Co-occurrence matrix (3D, averaged) | Autocorrelation | cm_auto_corr_3D_avg |
| Co-occurrence matrix (3D, averaged) | Cluster tendency | cm_clust_tend_3D_avg |
| Co-occurrence matrix (3D, averaged) | Cluster shade | cm_clust_shade_3D_avg |
| Co-occurrence matrix (3D, averaged) | Cluster prominence | cm_clust_prom_3D_avg |
| Co-occurrence matrix (3D, averaged) | Information correlation 1 | cm_info_corr1_3D_avg |
| Co-occurrence matrix (3D, averaged) | Information correlation 2 | cm_info_corr2_3D_avg |
| Co-occurrence matrix (3D, merged) | Joint maximum | cm_joint_max_3D_comb |
| Co-occurrence matrix (3D, merged) | Joint average | cm_joint_avg_3D_comb |
| Co-occurrence matrix (3D, merged) | Joint variance | cm_joint_var_3D_comb |
| Co-occurrence matrix (3D, merged) | Joint entropy | cm_joint_entr_3D_comb |
| Co-occurrence matrix (3D, merged) | Difference average | cm_diff_avg_3D_comb |
| Co-occurrence matrix (3D, merged) | Difference variance | cm_diff_var_3D_comb |
| Co-occurrence matrix (3D, merged) | Difference entropy | cm_diff_entr_3D_comb |
| Co-occurrence matrix (3D, merged) | Sum average | cm_sum_avg_3D_comb |
| Co-occurrence matrix (3D, merged) | Sum variance | cm_sum_var_3D_comb |
| Co-occurrence matrix (3D, merged) | Sum entropy | cm_sum_entr_3D_comb |
| Co-occurrence matrix (3D, merged) | Angular second moment | cm_energy_3D_comb |
| Co-occurrence matrix (3D, merged) | Contrast | cm_contrast_3D_comb |
| Co-occurrence matrix (3D, merged) | Dissimilarity | cm_dissimilarity_3D_comb |
| Co-occurrence matrix (3D, merged) | Inverse difference | cm_inv_diff_3D_comb |
| Co-occurrence matrix (3D, merged) | Inverse difference normalized | cm_inv_diff_norm_3D_comb |
| Co-occurrence matrix (3D, merged) | Inverse difference moment | cm_inv_diff_mom_3D_comb |
| Co-occurrence matrix (3D, merged) | Inverse difference moment normalized | cm_inv_diff_mom_norm_3D_comb |
| Co-occurrence matrix (3D, merged) | Inverse variance | cm_inv_var_3D_comb |
| Co-occurrence matrix (3D, merged) | Correlation | cm_corr_3D_comb |
| Co-occurrence matrix (3D, merged) | Autocorrelation | cm_auto_corr_3D_comb |
| Co-occurrence matrix (3D, merged) | Cluster tendency | cm_clust_tend_3D_comb |
| Co-occurrence matrix (3D, merged) | Cluster shade | cm_clust_shade_3D_comb |
| Co-occurrence matrix (3D, merged) | Cluster prominence | cm_clust_prom_3D_comb |
| Co-occurrence matrix (3D, merged) | Information correlation 1 | cm_info_corr1_3D_comb |
| Co-occurrence matrix (3D, merged) | Information correlation 2 | cm_info_corr2_3D_comb |
| Run length matrix (3D, averaged) | Short runs emphasis | rlm_sre_3D_avg |
| Run length matrix (3D, averaged) | Long runs emphasis | rlm_lre_3D_avg |
| Run length matrix (3D, averaged) | Low grey level run emphasis | rlm_lgre_3D_avg |
| Run length matrix (3D, averaged) | High grey level run emphasis | rlm_hgre_3D_avg |
| Run length matrix (3D, averaged) | Short run low grey level emphasis | rlm_srlge_3D_avg |
| Run length matrix (3D, averaged) | Short run high grey level emphasis | rlm_srhge_3D_avg |
| Run length matrix (3D, averaged) | Long run low grey level emphasis | rlm_lrlge_3D_avg |
| Run length matrix (3D, averaged) | Long run high grey level emphasis | rlm_lrhge_3D_avg |
| Run length matrix (3D, averaged) | Grey level non-uniformity | rlm_glnu_3D_avg |
| Run length matrix (3D, averaged) | Grey level non-uniformity normalized | rlm_glnu_norm_3D_avg |
| Run length matrix (3D, averaged) | Run length non-uniformity | rlm_rlnu_3D_avg |
| Run length matrix (3D, averaged) | Run length non-uniformity normalized | rlm_rlnu_norm_3D_avg |
| Run length matrix (3D, averaged) | Run percentage | rlm_r_perc_3D_avg |
| Run length matrix (3D, averaged) | Grey level variance | rlm_gl_var_3D_avg |
| Run length matrix (3D, averaged) | Run length variance | rlm_rl_var_3D_avg |
| Run length matrix (3D, averaged) | Run entropy | rlm_rl_entr_3D_avg |
| Run length matrix (3D, merged) | Short runs emphasis | rlm_sre_3D_comb |
| Run length matrix (3D, merged) | Long runs emphasis | rlm_lre_3D_comb |
| Run length matrix (3D, merged) | Low grey level run emphasis | rlm_lgre_3D_comb |
| Run length matrix (3D, merged) | High grey level run emphasis | rlm_hgre_3D_comb |
| Run length matrix (3D, merged) | Short run low grey level emphasis | rlm_srlge_3D_comb |
| Run length matrix (3D, merged) | Short run high grey level emphasis | rlm_srhge_3D_comb |
| Run length matrix (3D, merged) | Long run low grey level emphasis | rlm_lrlge_3D_comb |
| Run length matrix (3D, merged) | Long run high grey level emphasis | rlm_lrhge_3D_comb |
| Run length matrix (3D, merged) | Grey level non-uniformity | rlm_glnu_3D_comb |
| Run length matrix (3D, merged) | Grey level non-uniformity normalized | rlm_glnu_norm_3D_comb |
| Run length matrix (3D, merged) | Run length non-uniformity | rlm_rlnu_3D_comb |
| Run length matrix (3D, merged) | Run length non-uniformity normalized | rlm_rlnu_norm_3D_comb |
| Run length matrix (3D, merged) | Run percentage | rlm_r_perc_3D_comb |
| Run length matrix (3D, merged) | Grey level variance | rlm_gl_var_3D_comb |
| Run length matrix (3D, merged) | Run length variance | rlm_rl_var_3D_comb |
| Run length matrix (3D, merged) | Run entropy | rlm_rl_entr_3D_comb |
| Size zone matrix (3D) | Small zone emphasis | szm_sze_3D |
| Size zone matrix (3D) | Large zone emphasis | szm_lze_3D |
| Size zone matrix (3D) | Low grey level emphasis | szm_lgze_3D |
| Size zone matrix (3D) | High grey level emphasis | szm_hgze_3D |
| Size zone matrix (3D) | Small zone low grey level emphasis | szm_szlge_3D |
| Size zone matrix (3D) | Small zone high grey level emphasis | szm_szhge_3D |
| Size zone matrix (3D) | Large zone low grey level emphasis | szm_lzlge_3D |
| Size zone matrix (3D) | Large zone high grey level emphasis | szm_lzhge_3D |
| Size zone matrix (3D) | Grey level non-uniformity | szm_glnu_3D |
| Size zone matrix (3D) | Grey level non-uniformity normalized | szm_glnu_norm_3D |
| Size zone matrix (3D) | Zone size non-uniformity | szm_zsnu_3D |
| Size zone matrix (3D) | Zone size non-uniformity normalized | szm_zsnu_norm_3D |
| Size zone matrix (3D) | Zone percentage | szm_z_perc_3D |
| Size zone matrix (3D) | Grey level variance | szm_gl_var_3D |
| Size zone matrix (3D) | Zone size variance | szm_zs_var_3D |
| Size zone matrix (3D) | Zone size entropy | szm_zs_entr_3D |
| Distance zone matrix (3D) | Small distance emphasis | dzm_sde_3D |
| Distance zone matrix (3D) | Large distance emphasis | dzm_lde_3D |
| Distance zone matrix (3D) | Low grey level emphasis | dzm_lgze_3D |
| Distance zone matrix (3D) | High grey level emphasis | dzm_hgze_3D |
| Distance zone matrix (3D) | Small distance low grey level emphasis | dzm_sdlge_3D |
| Distance zone matrix (3D) | Small distance high grey level emphasis | dzm_sdhge_3D |
| Distance zone matrix (3D) | Large distance low grey level emphasis | dzm_ldlge_3D |
| Distance zone matrix (3D) | Large distance high grey level emphasis | dzm_ldhge_3D |
| Distance zone matrix (3D) | Grey level non-uniformity | dzm_glnu_3D |
| Distance zone matrix (3D) | Grey level non-uniformity normalized | dzm_glnu_norm_3D |
| Distance zone matrix (3D) | Zone distance non-uniformity | dzm_zdnu_3D |
| Distance zone matrix (3D) | Zone distance non-uniformity normalized | dzm_zdnu_norm_3D |
| Distance zone matrix (3D) | Zone percentage | dzm_z_perc_3D |
| Distance zone matrix (3D) | Grey level variance | dzm_gl_var_3D |
| Distance zone matrix (3D) | Zone distance variance | dzm_zd_var_3D |
| Distance zone matrix (3D) | Zone distance entropy | dzm_zd_entr_3D |
| Neighborhood grey tone difference matrix (3D) | Coarseness | ngt_coarseness_3D |
| Neighborhood grey tone difference matrix (3D) | Contrast | ngt_contrast_3D |
| Neighborhood grey tone difference matrix (3D) | Busyness | ngt_busyness_3D |
| Neighborhood grey tone difference matrix (3D) | Complexity | ngt_complexity_3D |
| Neighborhood grey tone difference matrix (3D) | Strength | ngt_strength_3D |
| Neighboring grey level dependence matrix (3D) | Low dependence emphasis | ngl_lde_3D |
| Neighboring grey level dependence matrix (3D) | High dependence emphasis | ngl_hde_3D |
| Neighboring grey level dependence matrix (3D) | Low grey level count emphasis | ngl_lgce_3D |
| Neighboring grey level dependence matrix (3D) | High grey level count emphasis | ngl_hgce_3D |
| Neighboring grey level dependence matrix (3D) | Low dependence low grey level emphasis | ngl_ldlge_3D |
| Neighboring grey level dependence matrix (3D) | Low dependence high grey level emphasis | ngl_ldhge_3D |
| Neighboring grey level dependence matrix (3D) | High dependence low grey level emphasis | ngl_hdlge_3D |
| Neighboring grey level dependence matrix (3D) | High dependence high grey level emphasis | ngl_hdhge_3D |
| Neighboring grey level dependence matrix (3D) | Grey level non-uniformity | ngl_glnu_3D |
| Neighboring grey level dependence matrix (3D) | Grey level non-uniformity normalized | ngl_glnu_norm_3D |
| Neighboring grey level dependence matrix (3D) | Dependence count non-uniformity | ngl_dcnu_3D |
| Neighboring grey level dependence matrix (3D) | Dependence count non-uniformity normalized | ngl_dcnu_norm_3D |
| Neighboring grey level dependence matrix (3D) | Dependence count percentage | ngl_dc_perc_3D |
| Neighboring grey level dependence matrix (3D) | Grey level variance | ngl_gl_var_3D |
| Neighboring grey level dependence matrix (3D) | Dependence count variance | ngl_dc_var_3D |
| Neighboring grey level dependence matrix (3D) | Dependence count entropy | ngl_dc_entr_3D |
| Neighboring grey level dependence matrix (3D) | Dependence count energy | ngl_dc_energy_3D |

Ⅱ. Names of the features used in the signatures

| **Table R1. Names of the features used in the signatures** | |
| --- | --- |
| Class | Features |
| No fusion-based features | |
| T1-weighted MR images | *cm_joint_max_3D_comb, cm_corr_3D_avg,*  *szm_szlge_3D, ngt_strength_3D* |
| T2-weighted MR images | *rlm_glnu_norm_3D_comb, dzm_hgze_3D,*  *dzm_zd_entr_3D, ngt_contrast_3D* |
| PET images | *ngt_complexity_3D, cm_joint_var_3D_avg,*  *ngl_hdlge_3D, ngt_coarseness_3D* |
| Image-level fusion based features | |
| T1-PET Image Fusion (0.1) | *rlm_rl_var_3D_comb, dzm_gl_var_3D,*  *rlm_lrlge_3D_avg, ngt_strength_3D* |
| T1-PET Image Fusion (0.2) | *rlm_lrlge_3D_avg, dzm_glnu_norm_3D,*  *dzm_sde_3D, ngt_contrast_3D* |
| T1-PET Image Fusion (0.3) | *rlm_rl_entr_3D_avg, dzm_lgze_3D,*  *dzm_zdnu_norm_3D, ngt_contrast_3D* |
| T1-PET Image Fusion (0.4) | *szm_zs_entr_3D, dzm_lgze_3D,*  *dzm_zdnu_norm_3D, ngt_contrast_3D* |
| T1-PET Image Fusion (0.5) | *dzm_hgze_3D, cm_clust_prom_3D_comb,*  *ngl_dc_energy_3D, szm_zs_entr_3D* |
| T1-PET Image Fusion (0.6) | *szm_hgze_3D, szm_sze_3D,*  *dzm_zd_entr_3D, rlm_rlnu_norm_3D_comb* |
| T1-PET Image Fusion (0.7) | *ngl_gl_var_3D, szm_sze_3D,*  *cm_joint_entr_3D_avg, ngt_strength_3D* |
| T1-PET Image Fusion (0.8) | *szm_szhge_3D, cm_diff_var_3D_avg,*  *dzm_gl_var_3D, ngt_strength_3D* |
| T1-PET Image Fusion (0.9) | *rlm_lrlge_3D_comb, cm_diff_var_3D_avg,*  *szm_gl_var_3D, ngt_coarseness_3D* |
| T2-PET Image Fusion (0.1) | *ngl_dc_var_3D, dzm_sde_3D,*  *dzm_gl_var_3D, ngt_contrast_3D* |
| T2-PET Image Fusion (0.2) | *dzm_glnu_norm_3D, ngl_hdlge_3D,*  *dzm_ldlge_3D, ngt_contrast_3D* |
| T2-PET Image Fusion (0.3) | *dzm_ldlge_3D, dzm_glnu_norm_3D,*  *dzm_sde_3D, ngt_contrast_3D* |
| T2-PET Image Fusion (0.4) | *cm_clust_tend_3D_avg, szm_szlge_3D,*  *dzm_zdnu_norm_3D, ngt_contrast_3D* |
| T2-PET Image Fusion (0.5) | *cm_clust_shade_3D_comb, szm_zs_entr_3D,*  *cm_info_corr2_3D_avg, ngl_gl_var_3D* |
| T2-PET Image Fusion (0.6) | *ngl_gl_var_3D, cm_auto_corr_3D_avg,*  *ngl_hdlge_3D, dzm_zd_entr_3D* |
| T2-PET Image Fusion (0.7) | *ngl_ldlge_3D, ngl_gl_var_3D,*  *ngl_hdlge_3D, dzm_zd_entr_3D* |
| T2-PET Image Fusion (0.8) | *dzm_zd_entr_3D, ngl_hdlge_3D,*  *szm_glnu_norm_3D, ngt_contrast_3D* |
| T2-PET Image Fusion (0.9) | *ngl_gl_var_3D, dzm_hgze_3D,*  *ngl_dc_entr_3D, dzm_zd_entr_3D* |
| Matrix-level fusion based features | |
| T1-PET Matrix Fusion | *cm_sum_entr_3D_avg, cm_diff_entr_3D_comb,*  *szm_szlge_3D, dzm_zd_entr_3D* |
| T2-PET Matrix Fusion | *ngl_dc_var_3D, szm_szhge_3D,*  *dzm_zdnu_norm_3D, ngt_contrast_3D* |
| Feature-level fusion based features | |
| T1-PET Feature Concatenation | *szm_lzlge_3D_T1, cm_joint_max_3D_comb_T1,*  *szm_szlge_3D_T1, ngt_coarseness_3D_PET* |
| T2-PET Feature Concatenation | *ngl_hdlge_3D_PET, dzm_glnu_norm_3D_T2,*  *ngl_ldlge_3D_T2, ngt_coarseness_3D_PET* |
| T1-PET Feature Average | *szm_szhge_3D, rlm_rl_entr_3D_comb,*  *szm_szlge_3D, ngt_strength_3D* |
| T2-PET Feature Average | *ngl_gl_var_3D, ngl_hdlge_3D,*  *ngl_ldlge_3D, ngt_strength_3D* |
| **Note:** For the class column, the number in the parentheses indicated the fusion weight of the MR images. | |

Ⅲ. Performance of radiomics signatures using 4-flod cross validation

| **Table R2. Performance of radiomics signatures using 4-flod cross validation** | | |
| --- | --- | --- |
| Class | Training dataset | Validation dataset |
| No fusion-based features | | |
| T1-weighted MR images | 0.8851± 0.0292 | 0.7381± 0.1668 |
| T2-weighted MR images | 0.8481± 0.0231 | 0.7351± 0.1135 |
| PET images | 0.8599± 0.0250 | 0.7937± 0.1204 |
| Image-level fusion based features | | |
| T1-PET Image Fusion (0.1) | 0.8805± 0.0347 | 0.8710± 0.0636 |
| T1-PET Image Fusion (0.2) | 0.8993± 0.0246 | 0.8175± 0.1088 |
| T1-PET Image Fusion (0.3) | 0.9049± 0.0348 | 0.8562± 0.1609 |
| T1-PET Image Fusion (0.4) | 0.8916± 0.0261 | 0.8056± 0.0836 |
| T1-PET Image Fusion (0.5) | 0.8922± 0.0209 | 0.7272± 0.1472 |
| T1-PET Image Fusion (0.6) | 0.9020± 0.0176 | 0.8046± 0.0588 |
| T1-PET Image Fusion (0.7) | 0.8818± 0.0354 | 0.6538± 0.1099 |
| T1-PET Image Fusion (0.8) | 0.8782± 0.0407 | 0.7857± 0.0554 |
| T1-PET Image Fusion (0.9) | 0.8711± 0.0299 | 0.7520± 0.1329 |
| T2-PET Image Fusion (0.1) | 0.8757± 0.0408 | 0.8175± 0.0902 |
| T2-PET Image Fusion (0.2) | 0.8833± 0.0364 | 0.8105± 0.1005 |
| T2-PET Image Fusion (0.3) | 0.8903± 0.0297 | 0.8095± 0.0994 |
| T2-PET Image Fusion (0.4) | 0.8820± 0.0420 | 0.8125± 0.1361 |
| T2-PET Image Fusion (0.5) | 0.8618± 0.0479 | 0.7937± 0.1833 |
| T2-PET Image Fusion (0.6) | 0.8560± 0.0448 | 0.7917± 0.1668 |
| T2-PET Image Fusion (0.7) | 0.8694± 0.0393 | 0.7834± 0.1781 |
| T2-PET Image Fusion (0.8) | 0.8480± 0.0272 | 0.7073± 0.0814 |
| T2-PET Image Fusion (0.9) | 0.8472± 0.0302 | 0.7143± 0.0625 |
| Matrix-level fusion based features | | |
| T1-PET Matrix Fusion | 0.8965± 0.0458 | 0.7351± 0.1841 |
| T2-PET Matrix Fusion | 0.8675± 0.0125 | 0.6974± 0.1260 |
| Feature-level fusion based features | | |
| T1-PET Feature Concatenation | 0.8965± 0.0360 | 0.7341± 0.1697 |
| T2-PET Feature Concatenation | 0.8750± 0.0388 | 0.6964± 0.1896 |
| T1-PET Feature Average | 0.8731± 0.0343 | 0.7639± 0.1802 |
| T2-PET Feature Average | 0.8811± 0.0293 | 0.7391± 0.1361 |
| **Note:** For the class column, the number in the parentheses indicated the fusion weight of the MR images. The AUC value was provided as mean± standard deviation. | | |
